# Editorial: Kidney transplant patients with COVID-19 infection

**DOI:** 10.3389/frtra.2024.1407254

**Published:** 2024-04-18

**Authors:** B. Handan Özdemir

**Affiliations:** Pathology Department, Faculty of Medicine, Baskent University, Ankara, Türkiye

**Keywords:** kidney, transplant, COVID-19, immunosuppression, vaccination, recipient outcomes

**Editorial on the Research Topic** Kidney transplant patients with COVID-19 infection

The topic “*Kidney Transplant Patients with COVID-19 Infection*.” continues to garner significant interest, reflecting the importance of understanding COVID-19's impact on kidney transplant recipients (KTRs). Despite vaccine and treatment availability, KTRs remain at risk for severe COVID-19 due to compromised immune systems, challenging the diagnosis of COVID-19 due to atypical symptoms and altered immune responses.

Obesity, hypertension, immunosuppressive (IS) therapy, and vaccination status significantly impact outcomes. The primary concern in KTRs was determining the optimal adjustment of IS therapy during COVID-19 infection. The goal was to balance promoting viral clearance with minimizing the risk of acute rejection and forming antibodies targeting the graft. Despite efforts to minimize IS, intensive care unit (ICU) mortality was high (44%), with one-year mortality reaching 67% (Jacq et al.). Vaccination reduces severe COVID-19 risk with lower mortality rates, especially with booster doses (Pinchera et al. and Jacq et al.). It must be noted that while mycophenolate-based therapies increase the severity risk of COVID-19, everolimus offers protection, and calcineurin inhibitors show no significant COVID-19 severity association (Pinchera et al.).

While minimizing IS therapy, the change in IS doses could increase the risk of acute rejection episodes and graft loss due to alloimmune injury. The incidence of rejection after COVID-19 varies across studies. Some studies reported a low incidence of graft loss among SARS-CoV-2-infected KTRs. In contrast, others showed no rejection after reducing or discontinuing antimetabolites and calcineurin inhibitors (CNIs). In the Jacq et al. cohort, no biopsy-proven acute rejection was observed following severe COVID-19, although one patient lost their graft within six months post-transplant. However, the absence of biopsies sometimes makes distinguishing between rejection and cortical necrosis challenging. The variability in study outcomes may be attributed to the small sample sizes or inadequate follow-up periods.

Early antiviral and monoclonal antibody intervention can also mitigate the severity of COVID-19 in KTRs (Pinchera et al.). Antiviral therapies, particularly early administration of Remdesivir and Molnupiravir, were associated with a reduced risk of developing severe disease. The data of Pinchera et al. align with previous studies, indicating the potential of early Remdesivir treatment to prevent severe COVID-19. Similarly, using Molnupiravir showed promise in reducing the risk of hospitalization and mortality in KTRs.

Regarding monoclonal antibodies, the study of Pinchera et al. found that early administration of Casirivimab/Imdevimab and Sotrovimab was associated with a lower risk of severe disease. While Casirivimab/Imdevimab showed no significant impact on the outcome, likely due to the small sample size, Sotrovimab effectively reduced the risk of poor outcomes related to severe disease. These findings are consistent with previous studies indicating lower hospitalization rates and mortality among solid organ transplantation (SOT) patients treated with monoclonal antibodies. The choice of monoclonal antibody should consider circulating variants of COVID-19, with Sotrovimab preferred against the Omicron BA.1 variant. However, reduced efficacy against the BA.2 variant has led to a decline in Sotrovimab use against this variant. Overall, the study of Pinchera et al. underscores the potential benefits of antiviral and monoclonal antibody treatments in mitigating severe COVID-19 outcomes in SOT patients, highlighting the importance of early intervention and tailored treatment strategies in this vulnerable population.

Identifying unique immune signatures in KTRs against SARS-CoV-2 provides mechanistic insights into the dysregulated immunity observed in immunosuppressed individuals with COVID-19. Fenninger et al. revealed that KTRs exhibit a diminished humoral immune response to SARS-CoV-2 but demonstrate a robust cellular immune response, possibly compensating for the impaired humoral response. These differences include a reduction in naïve T cells but an increase in effector T cells, a higher count of CD28+ CD4 effector memory T cells, and elevated CD8 T memory stem cells. Additionally, transplant patients exhibit lower levels of serum cytokine MIP-1β and a less diverse T cell receptor repertoire.

Vaccination is a crucial step to either ensure a mild course of COVID-19 disease or complete protection from it. However, IS treatments of KTRs prevent the achievement of protective immunogenicity after vaccination. Thus, to highlight the degree of protection of vaccines in KTRs, Körber et al. assessed cellular and humoral immunity and breakthrough infection rates in KTRs vaccinated with homologous and heterologous COVID-19 vaccination. They found that both homologous (hoVac) and heterologous (heVac) vaccine recipients developed robust T-cell responses after the third vaccination, regardless of the regimen. HeVac KTRs exhibited higher T-cell response rates and cytokine production than hoVac recipients after the second dose. However, after the third dose, T-cell response rates became comparable between the two groups. Additionally, heVac recipients showed higher serum neutralization capacities against the Omicron BA.5 variant. Furthermore, Körber et al. observed a correlation between T-cell responses and neutralizing antibody titers, suggesting an additional layer of protection. Factors like low estimated glomerular filtration rate and use of IS medications like mycophenolate mofetil were associated with lower antibody responses. The study concluded that continuous vaccination and monitoring of immune responses are crucial for protecting immunocompromised patients against COVID-19.

The COVID-19 pandemic has significantly impacted the management of KTRs and those with compromised immune systems. Questions persist regarding the safety and prognosis of kidney transplantation in individuals recovering from COVID-19. In the case report of Antal et al., they suggest that kidney transplantation may proceed earlier than recommended in vaccinated, asymptomatic individuals with mild COVID-19. Individualized risk assessment and careful consideration of immune status, vaccination history, and COVID-19 severity are crucial in decision-making for kidney transplantation post-COVID-19 infection.

Boosting early detection rates and enabling early treatment and remote monitoring of COVID-19 cases, especially in KTRs, has created a strong impetus for telemedicine. Zahradka et al. examined the impact of telehealth on the mortality rate of KTRs during the COVID-19 pandemic. The study indicates that KTRs referred to the transplant center early and receiving close monitoring had a lower risk of death from COVID-19. This early referral likely facilitated the timely administration of targeted treatment, including antiviral drugs and monoclonal antibodies, most effective within the initial viral replication phase. Adjustments to immunosuppression may also have contributed to improved outcomes. While the study doesn't evaluate vaccine effectiveness, it highlights the importance of vaccination in reducing infection rates among KTRs.

In conclusion, individualized risk assessment and early referral, coupled with vaccination, play pivotal roles in improving outcomes and reducing mortality rates among KTRs during COVID-19. Understanding immune responses post-vaccination and implementing continuous vaccination and monitoring protocols are crucial for protecting KTRs.

